# Biliary Complications Post Transjugular Intrahepatic Portosystemic Shunt in a Child With Portal Vein Cavernous Transformation

**DOI:** 10.7759/cureus.58525

**Published:** 2024-04-18

**Authors:** Johan S Lopera Valle, Brayan Muñoz-Caicedo, Julián A Muñoz Durán, José M Hidalgo Oviedo

**Affiliations:** 1 Department of Interventional Radiology, Hospital Universitario San Vicente Fundación, Medellín, COL; 2 Department of Radiology, Universidad de Antioquia, Medellín, COL; 3 Department of Interventional Radiology, Hospital Pablo Tobón Uribe, Medellín, COL

**Keywords:** portal hypertension, transjugular intrahepatic portosystemic shunt, biloma, leak, biliary injury, biliary obstruction

## Abstract

The transjugular intrahepatic portosystemic shunt is a rising interventional procedure with multiple indications and high technical success but with risks of biliary injuries, an underreported scenario. We present an 11-year-old patient with biliary injury with a leak, biloma formation, and biliary obstruction caused by the percutaneous procedure. Interventional radiology drainages addressed these complications by resolving the leak and biloma. These biliary complications in percutaneous procedures and their management are rarely reported in the medical literature, making their management not standard. We highlight drainage management and the importance of sharing it to add experience to this clinical scenario and encourage sharing cases with similar diagnoses.

## Introduction

The transjugular intrahepatic portosystemic shunt (TIPS) is a percutaneous procedure that connects the portal system and the central venous circulation through a liver parenchymal tract opened by metallic stents. It is an effective treatment for portal hypertension and gastrointestinal bleeding, among other indications [1]. Portal vein cavernous transformation challenges TIPS-making and increases the risk of complications and technical failure; however, technological advancements and multiple simultaneous vascular accesses during the TIPS procedure allow it to be done in such patients [2].

Complications for the TIPS can be broadly categorized as related to the procedure or the stent. For procedure complications, puncture to the biliary tree is generally well tolerated [3]. However, severe biliary injuries can occur during TIPS implantation and represent less than 1% of cases with a very low number of reports and outdated [4]. On the other hand, the TIPS stent by itself can compress the biliary tree with obstructive complications that can even require liver transplantation [5,6]. These biliary injuries are potential and underdescribed complications, even more so with the increasing and widespread use of noninvasive hepatobiliary procedures [7,8].

We report a case of biliary complications of the TIPS procedure in a child with portal vein cavernous transformation, intending to share the percutaneous management and good clinical results.

## Case presentation

An 11-year-old patient with a past medical history of thrombotic Behcet's disease and portal hypertension due to cavernous transformation of the portal vein had multiple bleeding episodes from the gastrointestinal tract, needing endoscopic treatment five times in the last two years. Recurrent bleeding, along with splenic sequestration and severe abdominal pain, prompted TIPS consideration.

**Figure 1:**
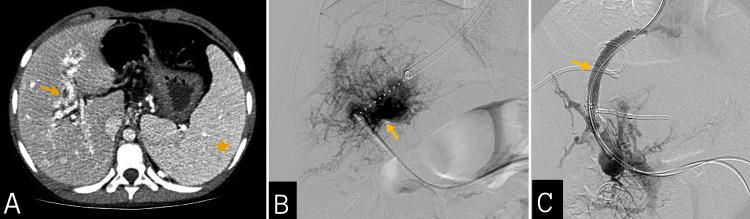
Abdominal contrast-enhanced computed tomography and portographies (A) Abdominal contrast-enhanced computed tomography shows the cavernous transformation of the portal vein (yellow arrow) and splenomegaly (yellow star). (B) Trans splenic portography shows the cavernous transformation of the portal vein (yellow arrow). The puncture was made from the middle hepatic vein to the portal circulation (white dots) using a hydrophilic vascular catheter in the portal cavernoma (yellow arrow) as a guide. (C) Successful portosystemic intrahepatic shunt implantation with the Viatorr stent (yellow arrow).

An abdominal contrast-enhanced computed tomography (CECT) showed a cavernous transformation of the portal vein. The esophageal varices and splenomegaly as signs of portal hypertension were also present. The multidisciplinary team decided to proceed with the TIPS procedure. The procedure was done using the Rösch-Uchida transjugular liver access set accessing through the right internal jugular vein and the trans splenic access for portography. The puncture was made from the middle hepatic vein to the portal circulation using a hydrophilic vascular catheter in the portal cavernoma as a guide. An 8 x 50 mm Viatorr stent was then inserted. The angiographic assessment confirmed the technical success of the portosystemic intrahepatic shunt, reducing portal venous pressure from 18 mmHg to 6 mmHg (Figure 1).

Seventy-two hours after the procedure, the patient's lab results indicated elevated total bilirubin levels from direct bilirubin and alkaline phosphatase, not accounted for by acute liver failure (Table 1).

**Table 1:** Laboratory results

Parameters	Patient values	Reference ranges
Hemoglobin	13.6 g/l	12-15 g/l
Hematocrit	42.5 %	41-54 %
Leukocytes	11350/mm^3^	4000-11000/mm^3^
Platelets	73000/mm^3^	150000-450000/mm^3^
Total bilirubin	1.4 mg/dl	0.2-1 mg/dl
Direct bilirubin	0.8 mg/dl	0-0.2 mg/dl
Alanine aminotransferase	8 U/l	7-56 U/l
Aspartate aminotransferase	27 U/l	5-40 U/l
Alkaline phosphatase	263 U/l	17-142 U/l
Gamma-glutamyl transferase	15 U/l	11-33 U/l
International normalized ratio	1.19	0.8-1.2

**Figure 2:**
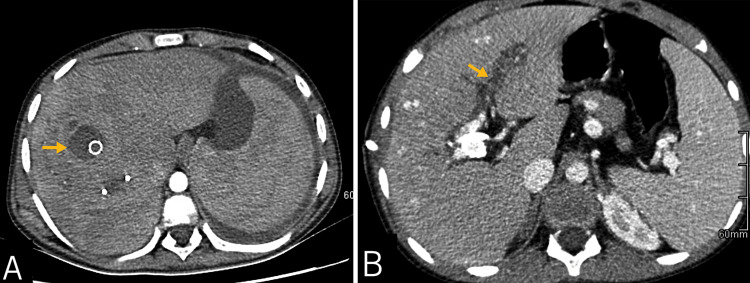
Abdominal contrast-enhanced computed tomography (A) It reveals a low-density collection around the transjugular intrahepatic portosystemic shunt (TIPS) stent (yellow arrow) (B) and dilation of the intrahepatic bile duct on the left side (yellow arrow).

A new abdominal CECT revealed a low-density collection around the TIPS stent and left-side intrahepatic bile duct dilation (Figure 2).

**Figure 3:**
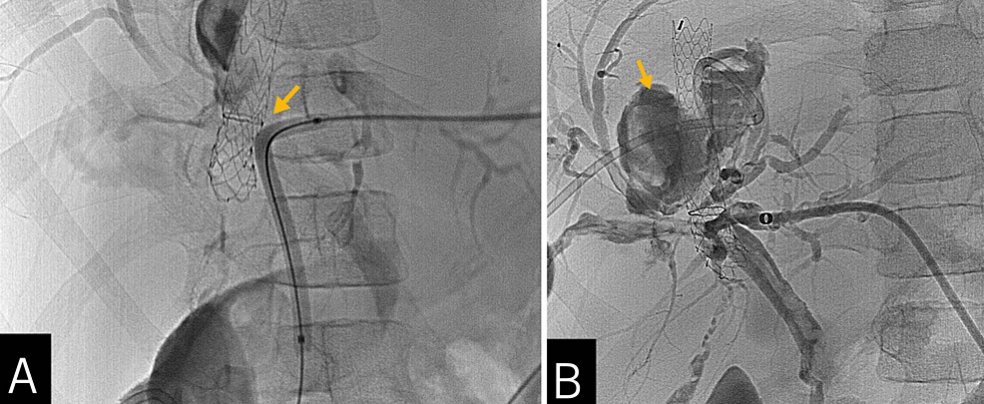
Percutaneous cholangiography 72 hours after the TIPS procedure (A) The bare portion of the transjugular intrahepatic portosystemic shunt (TIPS) stent (yellow arrow) caused external compression of the left hepatic duct near the confluence. Dilation with an angioplasty balloon was necessary for internal-external catheter placement. (B) Cholangiography reveals a significant contrast extraluminization from the right biliary tree (yellow arrow) that extends around the TIPS stent and communicates with the periportal venous collaterals. Findings were consistent with a right intrahepatic biliary injury with a leak, a biloma formation, and a biliary-venous fistula. There was no communication with the stent.

The patient underwent transparieto-hepatic cholangiography for bile duct drainage with an internal-external catheter from the left side. During catheter passage, external compression of the left hepatic duct near the confluence was noted due to the TIPS stent's bare portion. Dilation with a 5 x 80 mm angioplasty balloon was required. The final cholangiography revealed a significant collection that connected with the biliary tree around the stent, consisting of an intrahepatic biliary injury with a leak and biloma formation. The biloma was percutaneously drained, yielding bilious material (Figure 3).

**Figure 4:**
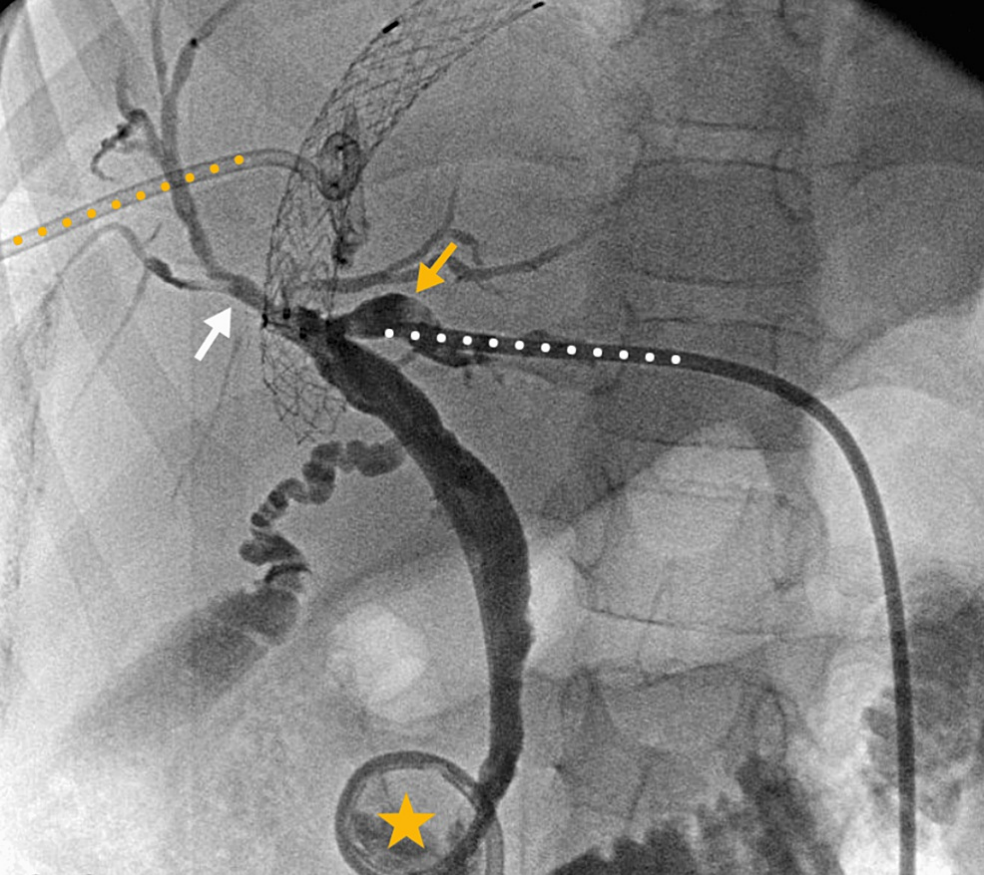
Four-week follow-up percutaneous cholangiography There was no biliary leak or collection, and improved dilation of the left bile duct (yellow arrow), which remained slightly larger than the right (white arrow). Bile duct drainage with an internal-external catheter was placed from the left side (white dots) to the duodenum (yellow star). An external catheter in the peri transjugular intrahepatic portosystemic shunt (TIPS) biloma is also shown (yellow dots).

Four weeks post-procedure, there was no biliary fistula or collection, and the left bile duct dilation had improved, remaining slightly larger than the right (Figure 4). After three months, the patient's symptoms of portal hypertension and cholestasis resolved (total bilirubin: 0.6 mg/dl, direct bilirubin: 0.18 mg/dl). However, biliary drainage remained necessary. There was no record of other follow-up visits to the institution.

## Discussion

TIPS is a procedure that percutaneously creates a channel between the portal system and a hepatic vein. The objective is to reduce the high portal pressure, which has many etiologies. Pediatric patients have the most frequent biliary atresia (21%); however, other causes exist, like portal thrombosis with cavernous transformation. TIPS is effective for decreasing portal hypertension and recurrent gastrointestinal bleeding. It is achieved successfully in more than 90% of cases. TIPS complications can be classified as related to the procedure or the stent and are around 3% but reported as 20%, with derived mortality at 0.84% [3,4,9,10].

In general, complications related to the TIPS procedure and percutaneous hepatic interventions potentially involve liver structures close to targeted areas, including the portal triad: portal vein, hepatic artery, and biliary ducts [11-14]. 

Inadvertent puncture to the biliary ducts is usually well tolerated; however, hemobilia and cholangitis could happen [3]. For this reason, biliary obstruction and dilatation are contraindications to the procedure, given the increased risk of injury and infection. However, it also occurs as a complication in patients with nondilated bile ducts, likely due to transect during the TIPS implantation, in which case severe biliary injuries, fortunately, are rare and represent less than 1% of cases [4].

So, when the injury to the biliary ducts occurs, it is important to establish the anatomy in imaging studies to define bile leakage and biloma formation where magnetic resonance imaging with a hepatospecific contrast agent is an emerging and useful imaging modality [15]. Once cleared, the biliary leak can be managed with nonsurgical means like percutaneous transhepatic biliary drainage under the principle of controlling the tract with a percutaneous catheter, deriving the bile from it, and promoting healing [11,12,16]. The limitations are mainly related to the psychological strain on the patients in long-term treatment that requires monitoring and multidisciplinary management when necessary [16]. In theory, the iatrogenic bile leaks without transection of the common biliary duct would be amenable to endoscopic treatment with biliary sphincterotomy or temporal biliary stenting to decrease the pressure gradient between the biliary tree and the duodenum, leading to preferential bile flow towards the duodenum. However, sphincterotomy can have risky complications in 15% of patients, especially in the pediatric [17]. If there is an injury to the common biliary duct associated with biloma, the combined endoscopic stent placement and radiological percutaneous drainage of the bile collection is indicated [18].

If a bare metal stent is used, communication between the TIPS and the biliary tree can occur [1]. In our case, there were no signs of communication between them. Besides, this fistula is virtually non-existent with the Viatorr endoprosthesis (Gore Medical, Newark, Delaware), given that it would be sealed by the expanded polytetrafluoroethylene covering (no biliary-shunt fistula) [1,14,19]. However, communication with the periportal collateral veins indicated a biliary-venous fistula. When hemobilia is associated with the fistula, embolization has been reported and considered needed [20]. In the presented patient, the material from the drainage was not hematic, and the fistula resolved with the biliary drain.

On the other hand, the stent-related complication of biliary obstruction is known due to compression at the TIPS passage site. In this situation, if applied, replacing the stent with a polytetrafluoroethylene-covered one or the biloma drainage has functioned well. However, in the short term (weeks), some patients continued deterioration with the final development of cirrhosis and secondary sclerosing cholangitis needing liver transplantation as a salvatory therapy [5,6]. Blood clots in the biliary tree can occur, producing obstruction that spontaneously resolves, an important differential diagnosis [3].

TIPS stent can cause bilomas by itself, secondary to bile duct compression or to the previously mentioned bile injury with a leak. In other words, a biloma can be a stent or procedure-related complication. The bile abdominal collection can be intrahepatic or extrahepatic, with an estimated incidence of 0.3-2% [4]. Management requires percutaneous, endoscopic, or surgical drainage in cases with collections greater than 4 cm; those smaller can be managed conservatively, except when infection is suspected [4]. In previous studies, biloma drains were removed after normalization of patient vital signs and lab values, drain flow less than 10 ml/24 h for three days, and complete resolution in imaging. Clinical and ultrasound follow-up to one, three, six, and 12 months have been recommended [16,18]. The differential diagnoses of bilomas are cysts, hematomas, loculated ascites, and seromas [4].

This case illustrates the complications of a pediatric patient with an indication of TIPS implantation due to portal cavernous transformation with increased pressure gradient and repeated gastrointestinal hemorrhage. The TIPS implantation was performed successfully with an adequate decrease in the pressure gradient. However, there was an intrahepatic biliary injury with leaking and biloma formation managed with drainage. Also, biliary obstruction caused by the TIPS stent was detected early in the post-procedure period and managed with bilioplasty and drain implantation. The challenges during TIPS placement in this patient with complex vascular anatomy may have contributed to the complications. However, the patient improved and had favorable outcomes.

Finally, like case reports, ours has many limitations, like patient selection bias, single institution management, lack of comparator interventions, non-long-term follow-up, and non-generalizable results. However, in searching for similar cases in the medical field, there is little evidence about this specific entity, which makes it a valuable experience to share. More studies are needed to establish the optimal treatment options.

## Conclusions

Iatrogenic biliary duct injuries are underdescribed and rare but latent risks with hepatic percutaneous interventions, even biopsies. With technological advancements and increased access to the TIPS procedure, complications will also increase, making it necessary to consider these low-frequency scenarios. When a biliary injury or obstruction occurs, the management objectives are to divert the bile to decrease end-organ complications, primarily in the liver. In our case, percutaneous drainage was effective in normalizing liver function, closing the fistula, drainage of the biloma, and surpassing the obstructive biliary effect of the TIPS stent, but the evidence is scarce, and more studies are necessary to standardize the best medical practice.
